# Interceptor® long-lasting insecticidal net: phase III evaluation over three years of household use and calibration with Phase II experimental hut outcomes

**DOI:** 10.1186/s13071-016-1490-9

**Published:** 2016-04-13

**Authors:** Patrick Tungu, Matthew Kirby, Robert Malima, William Kisinza, Stephen Magesa, Caroline Maxwell, Benard Batengana, Olivier Pigeon, Mark Rowland

**Affiliations:** National Institute for Medical Research, Amani Medical Research Centre, Muheza, Tanzania; London School of Hygiene and Tropical Medicine, WC1E 7HT London, UK; Walloon Agricultural Research Centre, Agriculture and Natural Environment Department, Rue du Bordia, 11 B-5030 Gembloux, Belgium

**Keywords:** Long-lasting insecticidal net, LLIN, *Anopheles gambiae*, Tanzania, Randomised controlled trial, Alpha-cypermethrin

## Abstract

**Background:**

Long-lasting insecticidal nets (LN) are an effective tool for malaria prevention. The World Health Organization Pesticide Evaluation Scheme has established evaluation criteria to facilitate registration for public use. A household randomised trial was conducted in Tanzania according to WHOPES Phase III procedures to evaluate the alpha-cypermethrin coated Interceptor® LN (BASF) over three years’ use. Outcomes were calibrated against results of Phase II experimental hut trials.

**Methods:**

Interceptor LN (200 mg/m^2^ alpha-cypermethrin) and conventionally treated nets CTN (40 mg/m^2^ alpha-cypermethrin) were randomly distributed to 934 households. At 6-monthly intervals, household surveys recorded net use, durability, adverse effects, user acceptance and washing practices. Concurrently, 30 nets of each type were collected and tested for knock-down and kill of *Anopheles gambiae* mosquitoes in cone and tunnel bioassays. Alpha-cypermethrin content of nets was assessed annually.

**Results:**

At 12 months 97 % of Interceptor LN met the efficacy criteria by cone or tunnel test; this pass rate declined to 90 % at 24 months and 87 % at 36 months. In contrast only 63 % of CTN met the efficacy criteria at 12 months, 14 % at 24 months and 0 % at 36 months. The alpha-cypermethrin content at 36 months on Interceptor LN was 20 % (42 ± 13 mg/m^2^) of the initial content but on CTNs only 4 % (1.3 ± 1.6 mg/m^2^) remained. Interceptor LN was reported to be used year-round and washed 4.3 times/year. A few recalled facial tingling during the first days of use but this did not deter usage. The average number of holes at 36 months was 18, hole area per net was 229 cm^2^ and hole index was 332. Insecticide content and cone bioefficacy of LN and CTN after 36 months’ use were similar to that of LN and CTN used in earlier Phase II hut trials, but while the 20 times washed LN tested in experimental huts gave adequate personal protection the 20 times washed CTN did not.

**Conclusions:**

More than 80 % Interceptor LN fulfilled the WHOPES Phase III criteria at 36 months and thus the LLIN was granted full WHO recommendation. Phase III outcomes at 36 months were anticipated by Phase II outcomes after 20 standardized washes.

## Background

Long-lasting insecticidal nets (LLIN) that repel or kill mosquitoes that make contact with the netting are the primary method of preventing malaria in many countries of Africa south of the Sahara and Asia [1–3]. The retention of this biological activity, through 20 washes and 3 years of field use *without need for re-treatment*, is ultimately what defines and distinguishes a long-lasting insecticidal net (LLIN) from a conventionally treated net (CTN) [4, 5]. Preservation of bio-efficacy is achieved during the manufacturing through one of three treatment processes: a) the active ingredient is incorporated into the synthetic fibre materials before the yarn is extruded; b) the extruded yarn is coated with insecticide and polymer binding agent before the nets are sewn; c) pre-sewn nets are mechanically sprayed with the insecticide plus binder. Formal evaluations of LLIN started more than a decade ago [6, 7]. More recently the WHO Global Malaria Programme has urged national malaria control programmes to purchase, promote and scale-up the coverage of LLINs [4], effectively phasing out CTN that require multiple re-treatments during the lifetime of use. Today, the World Health Organisation reports that almost half of the African population at risk from malaria has access to insecticide treated nets (mainly LLIN) in the home and an estimated 44 % were sleeping under treated nets compared to 2 % in 2004 [8]. Several brands of LLIN are recommended by WHOPES. One of these is Interceptor® LN (BASF Corporation, Germany) [9], which even after 20 standardised washes demonstrates high killing effect (> 75 %) and personal protection (> 75 %) against malaria vectors in Phase II experimental hut trials [9, 10]. However, less is known of the longevity, physical integrity, attrition rate, persistence of bio-efficacy and insecticide content of LLIN under household conditions. For donors and procurement organizations, such information is vital to the planning of LLIN distribution and replacement campaigns.

Interceptor LN nets contain a textile auxiliary Fendozin® (BASF) as a finishing product that binds the alpha-cypermethrin insecticide to the polyester fibres in a resin-based polymer coating [9, 10]. This coating can withstand multiple washes and yet allows the slow release of the alpha-cypermethrin to the net surface where it rapidly knocks down and kills mosquitoes as they make contact with the net.

Some field studies of Interceptor® LN have shown encouraging efficacy and acceptability outcomes over 1–3 years of use [11–14]. In Liberia, a prospective study showed a low rate of insecticide loss and high acceptability of Interceptor LN [11]; however, these outcomes were measured for only 1 year post-distribution. In north-eastern India, two groups of three [12] and six [14] villages received Interceptor LN in field trials, which resulted in large reductions in vector mosquito population densities. However, in these studies the intervention villages were compared with villages that received untreated nets or focal spraying of DDT instead of conventionally treated nets. As such, neither the study design nor the outcome measure (reduction of vector abundance) satisfies the requirements of a WHOPES Phase III trial or WHOPES criteria for full recommendation. A full WHOPES recommendation is only granted after demonstrating that the candidate LLIN still meets specific efficacy criteria after 3 years of regular household use in clearly defined phase III trials [5, 15].

The overall objective of this study was to carry out a Phase III evaluation of Interceptor LN in line with WHOPES guidelines and procedures to determine their efficacy, longevity, integrity, wash resistance and household acceptability under field conditions. The specific objectives were a) to evaluate Interceptor LN in terms of biological efficacy at 7 time points over 36 months in comparison with conventional alpha-cypermethrin treated nets used under similar field conditions, b) to determine chemical content at annual intervals up to 3 years of use, c) to monitor net integrity, d) to assess household acceptance and use of Interceptor LN, and e) to calibrate the outcomes of Phase III household trials with outcomes of Phase II experimental hut trials.

## Methods

### Study area

**Fig. 1:**
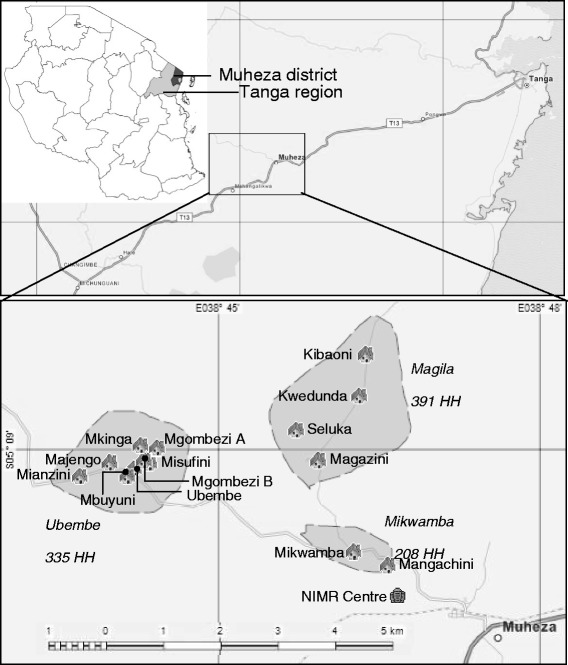
Location of study villages and hamlets within Muheza district, Tanzania

The study site was comprised of 3 villages containing 15 hamlets in Muheza district, Tanga region, northeast Tanzania (Fig. 1). The household demographic survey and baseline census was conducted in 2008. Magila village consists of 5 hamlets (Kibaoni, Kwedunda, Magazini, Potwe, Seluka), 391 houses and a population of 2,959. Ubembe village consists of 8 hamlets (Mianzini, Majengo, Ubembe, Misufini, Mbuyuni, Mgombezi A and B, Mkinga), 335 households and a population of 1,478. Mikwamba village consists of 2 hamlets (Mikwamba, Mangachini), 208 households and a population of 943.

The area experiences a long rainy season between April and August and a short rainy season between December and January. During the rainy seasons *Anopheles gambiae* sensu lato predominates. *An. funestus* becomes more common in the dry season. The area has been shown in the past to have high entomological inoculation rates, estimated between 300 and 1,000 infective bites per person per year [16].

### Study design

A 3 year community randomized trial was conducted with the household as the unit of randomization and with the mosquito nets as the unit of observation. The efficacy of Interceptor LN was monitored over 3 years of continuous use. Conventionally treated nets were used for comparative purposes. Initially it was proposed to replace the CTNs with LNs 1 year into the trial as recommended in the WHOPES 2005 guidelines for laboratory and field testing of LN [15]. Unfortunately, thieves raided the store in which the replacement nets were stockpiled and all the nets were stolen. After review the decision was made to continue monitoring the CTN until a randomly selected net failed to meet the cut-off bioassay criteria (see section *Insecticide bioassay efficacy of nets*), at which point all the nets from that household were replaced with LN. This study pre-dated the 2013 revised WHOPES guidelines for laboratory and field testing of LN, which recommended that a candidate LN is field evaluated against an existing WHO-recommended LN rather than a CTN [5].

### Household randomisation

**Fig. 2:**
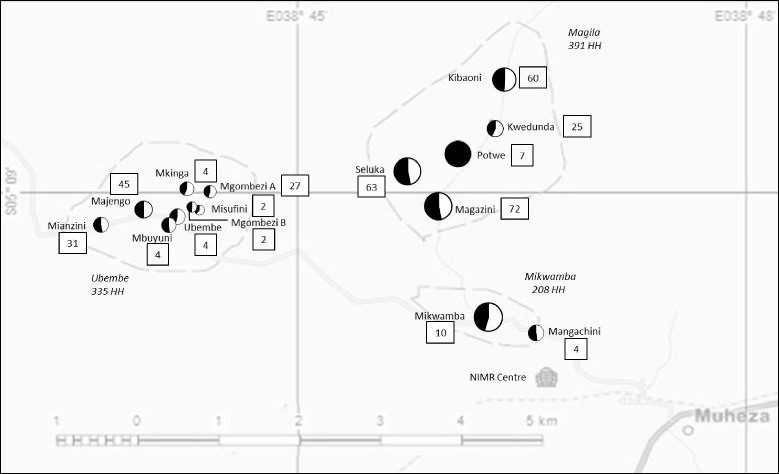
Distribution of study nets within the 15 hamlets. Pie chart diameter is proportional to the total number of nets distributed in each hamlet (black = LN, white = CTN). Numbers in squares are the total nets (LN + CTN) destructively sampled from each hamlet

A pre-distribution baseline census collected details of residents including the number of sleeping places per household, sizes of beds, net ownership and net usage. A household was defined as a group of related or unrelated persons living together in the same dwelling, acknowledging one adult as the household head. Each household was given a unique identification number and the house was physically labeled with this number to facilitate revisits. The household number was used to randomly allocate the Interceptor LNs or CTNs to selected households, stratified by hamlet so that both net types were well represented within each hamlet (Fig. 2). The allocation of nets to each household was dependent on the number of sleeping places. Every bed or sleeping place had to be covered by a net in all hamlets to ensure the community was adequately protected.

### Net treatment and distribution

Interceptor® LNs and untreated polyester nets of 75 denier were supplied by BASF Corporation (Ludwigshaven, Germany) in a range of sizes. Interceptor LN was treated with alpha-cypermethrin (coated onto filaments) at a target dose of 6.7 g AI/kg of netting material for 75-denier yarn, corresponding to 200 mg alpha-cypermethrin per m^2^ of polyester fabric (with a tolerance limit of ±25 %). Polyester nets of the same denier and supplier were treated individually at the NIMR Amani Medical Research Centre using an aqueous solution of alpha-cypermethrin (Fendona 10SC, BASF), with volume dependent on the size of the net using the formula of Pleass et al. [17] to achieve a target dosage of 40 mg/m^2^. Nets were laid flat over plastic sheeting to dry in shaded conditions and rolled over periodically until the insecticide solution had dried. A unique code number was stenciled onto each net using a permanent marker. Nets were also marked with a cross in water-soluble ink for the assessment of washing practices.

A total of 1,953 Interceptor LN and 1,593 CTN were distributed to the selected households in 2008. The household number was used as the unit to randomly allocate the Interceptor and CTNs. The distribution teams and the recipients were blinded to the identity of the nets received by each household. Individual households received either Interceptor LNs or CTNs and not a mix of types. Enough nets were distributed to cover all sleeping spaces in the house. Householders were informed about the need for reporting adverse effects during net use, as well as advised on appropriate use and maintenance of nets. Assistance in hanging up the nets over the sleeping area was given where needed.

### Household surveys and net sampling

Thirty nets of each type were sampled at baseline (pre-distribution) and during cross sectional surveys of households carried out after 6, 12, 18, 24, 30 and 36 months of field use. The 60 households sampled per survey were selected randomly from the household ID master list. The selected households received a replacement LN and were removed from the study. Figure 2 shows the number of nets sampled from each hamlet. At the time of collection, a questionnaire was applied to assess current net use, acceptability, washing practices and any adverse effects.

### Net integrity and durability

Net integrity and durability surveys were carried out for Interceptor LN at baseline and 6, 12, 18, 24, 30 and 36 months post-distribution and for CTNs at baseline, 6 and 12 months post-distribution. The nets were hung over a wooden frame and scored for the size and distribution of holes, repairs (stitches, knots and patches) and open/failed seams. Cleanliness assessment was done simultaneously and nets categorised according to grade of cleanliness/dirtiness.

Hole sizes were categorised as size 1 – smaller than a thumb, size 2 – larger than a thumb but smaller than a fist, size 3 – larger than a fist but smaller than a head, size 4 – larger than a head. Hole index, hole area and hole circumference were estimated using the following formulae:

$$ \mathrm{Hole}\ \mathrm{index} = \left(\mathrm{no}.\ \mathrm{of}\ \mathrm{size}\ 1\ \mathrm{holes} \times 1\right) + \left(\mathrm{no}.\ \mathrm{of}\ \mathrm{size}\ 2\ \mathrm{holes} \times 23\right) + \left(\mathrm{no}.\ \mathrm{of}\ \mathrm{size}\ 3\ \mathrm{holes} \times 196\right) + \left(\mathrm{no}.\ \mathrm{of}\ \mathrm{size}\ 4\ \mathrm{holes} \times 578\right). $$

$$ \mathrm{Hole}\ \mathrm{area} = \left(\mathrm{no}.\ \mathrm{of}\ \mathrm{size}\ 1\ \mathrm{holes} \times 0.25\uppi \right) + \left(\mathrm{no}.\ \mathrm{of}\ \mathrm{size}\ 2\ \mathrm{holes} \times 9\uppi \right) + \left(\mathrm{no}.\ \mathrm{of}\ \mathrm{size}\ 3\ \mathrm{holes} \times 25\uppi \right). $$

$$ \mathrm{Hole}\ \mathrm{circumference} = \left(\mathrm{no}.\ \mathrm{of}\ \mathrm{size}\ 1\ \mathrm{holes} \times 1\uppi \right) + \left(\mathrm{no}.\ \mathrm{of}\ \mathrm{size}\ 2\ \mathrm{holes} \times 6\uppi \right) + \left(\mathrm{no}.\ \mathrm{of}\ \mathrm{size}\ 3\ \mathrm{holes} \times 10\uppi \right) $$

In the formula for hole index, the multipliers used assume that the hole size equates to the mid-point of the range for each hole size category using the method described by WHO [18]. The formulae for calculating the hole area and hole circumference was based on the area and circumference of a circle: size 1 holes were of 0–2 cm diameter (midpoint = 1 cm). Size 2 holes were of 2–10 cm diameter (midpoint = 6 cm). Because no size 4 holes were observed and few holes categorised as size 3 were wider than 10 cm, the average diameter of size 3 holes was set at the lower limit of 10 cm diameter. Thus the estimate of hole area gives a slightly more conservative value when compared to the hole index. Hole circumference was included as it might be the more biologically relevant indicator: mosquitoes walking or skipping across the surface of net must encounter the edge of a hole before penetrating the net.

### Insecticide bioassay efficacy of nets

Thirty Interceptor LNs and 30 CTNs were sampled at baseline and at 6, 12, 18, 24, 30 and 36 months post-distribution. Five netting pieces (25 cm × 25 cm) were cut from the five panels of each net in accordance with WHOPES guidelines [15]. Cone bioassay tests were carried out on the netting pieces at the NIMR Amani Centre using 2–5 day old, unfed, female *An. gambiae* (*s.s*.) (Kisumu strain). Twenty mosquitoes were exposed in 4 replicates of 5 mosquitoes to 1–4 pieces of each net (total of 80 mosquitoes per net) for 3 min in standard WHO plastic cones; the 5^th^ piece nearest the point of abrasion where the net is tucked under the mattress was excluded as recommended by WHOPES. After exposure the mosquitoes were held in paper cups at 26 °C and 80 % relative humidity with access to cotton wool soaked in 10 % glucose solution. Knockdown was recorded 1 h after exposure and mortality was recorded after 24 h. When knockdown was < 95 % and mortality was < 80 %, the net was subjected to a tunnel test [15]. Only the net piece closest to average mortality of the net was used for the tunnel test. Any net meeting the cone criteria of ≥ 80 % mortality or ≥ 95 % knockdown or tunnel test criteria of ≥ 80 % mortality or ≥ 90 % blood-feeding inhibition was considered to have met the WHOPES criteria.

### Chemical analysis

From each of the 30 LN and 30 CTN sampled at baseline and surveys at 12, 24 and 36 months, five additional pieces of netting (30 cm × 30 cm) were cut for chemical analysis. As before, the pieces were cut from the five panels of each net and the piece closest to the mattress line was excluded as per WHOPES guidelines. All Interceptor pieces and the baseline and 12 month CTN pieces were sent to the WHO-collaborating Centre Wallon de Recherches Agronomiques (CRA-W) in Belgium for chemical analysis. The net pieces from each individual net was pooled, cut into small pieces and homogenized, and alpha-cypermethrin was extracted from an aliquot by heating under reflux with tetrahydrofuran in accordance with the CIPAC method for alpha-cypermethrin in coated LNs. Dioctyl phthalate was added as an internal standard; alpha-cypermethrin content of each individual net was determined using gas chromatography with flame ionization detection (GC-FID). Pieces from CTN at 24 and 36 months were analysed by high-performance liquid chromatography at The London School of Hygiene and Tropical Medicine (LSHTM) using the method described by Yates et al. [19].

### Data analysis

Data were double-entered into Microsoft Access 2007 and analysed in STATA version 10.1 Proportional data such as the 1 h knockdown and 24 h mortality was transformed using square root arc sign method before analysis. Categorical data was analysed using Chi-square, and assessment of net efficacy over successive surveys was analysed using Chi-square tests for trend. Continuous data was analysed using Wilcoxon rank sum test where the data was not normally distributed.

### Ethical considerations and approval

Ethical clearance was received from the Medical Research Coordination Committee of the National Institute of Medical Research, which is the National Ethics Committee of the Ministry of Health in Tanzania. The project also obtained ethical clearance from the Research Ethics Committees of LSHTM and WHO.

## Results

### Household surveys

A total of 3,546 sleeping places were identified across all study hamlets in the baseline survey, and 77 % of these contained beds. Most beds were size 5′ × 6′ (2,066) or 6′ × 6′ (660). Nets of appropriate size were given to cover all sleeping places. During the net-sampling cross sectional surveys, households were asked about house characteristics, net use and net washing practices. The majority of houses had palm thatched roofs (range between surveys 50–64 %), though corrugated iron was also common. Most householders were farmers (range 43–97 %) and most (65–79 %) had received 7 or more years of primary school education but less than 10 % had received secondary or further education. Over a third of households lived on less than $1 per day; the highest salary recorded was only $3 per day and the mean income was $1.75 US per day.

### Net use and washing

Reported use of both types of net was high throughout the study. At 12, 24 and 36 months post-distribution, all respondents indicated using their nets year round and every night. The placement of nets appeared to provide corroboration; 98 % (127/130) of Interceptor LN were found hung above beds and the remaining 3 LNs were observed suspended over floor mattresses. Similarly in the CTN group 99 % (118/119) of nets were seen hung over a bed.

Interceptor LNs and CTNs were washed on average 4.3 times per year. Despite this it was observed that 70 % of Interceptor LN and 77 % of CTN had accumulated some dirt after 6 months, and this proportion increased after 12 months (Table 1). After 36 months only 10 % of the Interceptor LNs were scored as clean and 27 % were scored as very dirty. No differences were reported between the washing practices of families using Interceptor LN and families using CTN. Virtually all respondents reported washing their nets in cold water. Nets were soaked by 20–37 % of respondents; soaking times ranged from 10 min to 2 h. Nets were reported washed using commercial bar soap (53–62 %), commercial detergent powder (17–27 %) or both (8–30 %). Most nets (68–90 %) were rinsed after washing and most (75–95 %) were dried outside. No one reported rubbing nets against rocks or stones during washing.

**Table 1:** Washing frequency and net appearance

	Interceptor LN						alpha-cypermethrin CTN					
			% general aspect of nets						% general aspect of nets			
Survey (month)	No. nets	Mean no. of washes^a^	Clean	Slightly dirty	Dirty	Very dirty	No. nets	Mean no. of washes^a^	Clean	Slightly dirty	Dirty	Very Dirty
0	30	0	100	0	0	0	30	0	100	0	0	0
6	30	3	30	40	30	0	30	4	23	47	27	3
12	30	3	13	30	47	10	30	2	20	27	40	13
18	30	2	3	0	97	0	-	-	-	-	-	-
24	30	1	23	0	73	4	-	-	-	-	-	-
30	30	2	10	37	43	10	-	-	-	-	-	-
36	30	2	10	20	43	27	-	-	-	-	-	-

^a^Mean number of washes during the six monthly periods

### Physical integrity

The baseline survey found no holes or open seams on any of the sampled Interceptor LNs or CTNs (Tables 2 and 3). After 6 months, 63 % of Interceptor LN and 83 % of CTN had at least one hole; these were mainly of size 1 and the mean number of holes was only 5 per net for Interceptor LN and 9 for CTN. By 24 months, 83 % of Interceptor LN had at least one hole and the mean number of holes per net was 22. After 36 months, the percentage of nets with at least one hole and the mean number of holes per net appeared to have not increased relative to the 24 month survey. From the 6th month survey onwards the majority of holes were size 1, approximately a quarter were size 2 and a minority were size 3. The vast majority of holes were always to be found in the lowest section of the net, at body level, where the net is tucked under the mattress (if present). The number and the position of holes did not differ between net types (both types being of the same 75 denier material). The physical integrity of the Interceptor nets deteriorated between 12 and 24 months with respect to hole index (Wilcoxon rank sum test *Z* = -2.797, *P* = 0.005), hole area (*Z* = -2.797, *P* = 0.005) and hole circumference (*Z* = -2.827, *P* = 0.005) but between 24 and 36 months no further deterioration was evident (hole index *Z* = -0.296, *P* = 0.77; hole area *Z* = -0.222, *P* = 0.82; hole circumference *Z* = -0.015, *P* = 0.99) (Table 4).

**Table 2:** Physical condition of Interceptor LN and CTN by survey round – holes by size category;

	Interceptor LN						alpha-cypermethrin CTN					
Survey (month)	No. nets	Mean (SD) holes/net	% holes by size category				No. nets	Mean (SD) holes/net	% holes by size category			
			1	2	3	4			1	2	3	4
0	30	0 (0)	0	0	0	0	30	0 (0)	0	0	0	0
6	30	5 (9)	64	26	10	0	30	9 (20)	75	22	3	0
12	30	6 (9)	71	16	13	0	30	11 (18)	72	22	6	0
24	30	22 (23)	68	25	7	0	-	-	-	-	-	-
36	30	18 (20)	67	27	6	0	-	-	-	-	-	-

**Table 3:** Physical condition of Interceptor LN and CTN by survey round – holes by distribution

	Interceptor LN							alpha-cypermethrin CTN						
Survey (month)	No. nets	% nets with ≥ 1 hole	% holes by distribution^a^			Mean no. of open seams	% nets with any repairs	No. nets	% nets with ≥ 1 hole	% holes by distribution^a^			Mean no. of open seams	% nets with any repairs
			Lower	Upper	Roof					Lower	Upper	Roof		
0	30	0	0	0	0	0	0	30	0	0	0	0	0	0
6	30	63	73	18	9	0	0	30	83	93	4	2	0	0
12	30	60	75	11	14	0.1	3	30	67	79	16	5	0.2	7
24	30	83	84	10	6	2.2	3	-	-	-	-	-	-	-
36	30	83	70	17	13	1.4	20	-	-	-	-	-	-	-

^a^Location of holes: lower = lower half of side panels; upper = upper half of side panels; roof = top panel

**Table 4:** Physical integrity – comparison of estimates of the average hole index, hole area and hole circumference for a) Interceptor LN; b) alpha-cypermethrin CTN

a)	Interceptor LN								
Survey (month)	Hole index			Hole area (cm^2^)			Hole circumference (cm)		
	Mean (1SD)	Median (IQR)	Geometric mean	Mean (1SD)	Median (IQR)	Geometric mean	Mean (1SD)	Median (IQR)	Geometric mean
0	0 (0)	0 (0)	0	0 (0)	0 (0)	0	0 (0)	0 (0)	0
6	139 (351)	2 (0–83)	7	83 (193)	2 (0–69)	6	59 (113)	6 (0–48)	8
12	170 (630)	2 (0–68)	7	88 (263)	1 (0–75)	6	54 (118)	5 (0–65)	7
24	442 (696)	78 (3–533)	46	282 (393)	84 (3–404)	37	194 (240)	63 (11–326)	45
36	332 (442)	126 (30–549)	70	229 (283)	102 (33–346)	60	162 (187)	127 (36–212)	54
b)	alpha-cypermethrin CTN								
Survey (month)	Hole index			Hole area (cm^2^)			Hole circumference (cm)		
	Mean (1SD)	Median (IQR)	Geometric mean	Mean (1SD)	Median (IQR)	Geometric mean	Mean (1SD)	Median (IQR)	Geometric mean
0	0 (0)	0 (0)	0	0 (0)	0 (0)	0	0 (0)	0 (0)	0
6	121 (324)	5 (1–51)	12	91 (257)	4 (1–60)	10	73 (186)	16 (4–53)	15
12	205 (458)	8 (0–173)	14	134 (253)	6 (0–87)	12	95 (161)	19 (0–76)	14
24	-	-	-	-	-	-	-	-	-
36	-	-	-	-	-	-	-	-	-

### Analysis of chemical content

**Fig. 3:**
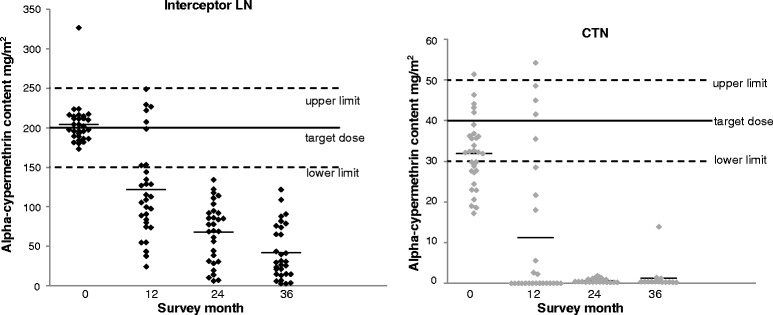
Alpha-cypermethrin content (mg AI/m^2^) on individual Interceptor® LN and CTN samples at baseline and after 12-monthly intervals of field use. Mean concentrations for each time point are indicated by the thin horizontal lines. The target dose and upper and lower limits are for alpha-cypermethrin content at baseline indicated as solid and dashed lines

At baseline the mean concentration of alpha-cypermethrin was 204 mg/m^2^ for Interceptor LN and 32 mg/m^2^ for CTN (Fig. 3). These values were within 25 % of the target dosages (200 and 40 mg/m^2^ respectively). The mean concentration of alpha-cypermethrin in the Interceptor LNs had decreased to 117 mg/m^2^ after 12 months, to 68 mg/m^2^ after 24 months and to 42 mg/m^2^ after 36 months (Fig. 3). The mean concentration of alpha-cypermethrin in the CTNs was 9.6 mg/m^2^ after 12 months’ field use, 0.7 mg/m^2^ after 24 months and 1.3 mg/m^2^ after 36 months. At some time points a difference was apparent in insecticide concentration between nets which passed the bioassay criteria (≥80 % mortality) and those which failed it: among the CTN at 12 months the mean concentration was 15.8 mg/m^2^ for those which passed and just 7.6 mg/m^2^ for those which failed; among the LN at 24 months the mean concentration was 74.5 mg/m^2^ for those which passed but only 55.1 mg/m^2^ for those which failed. However, it is interesting to note that 40–50 % of mosquitoes were still being knocked down and killed by the CTN sampled after 36 months despite very low insecticide concentrations on the nets.

### Net efficacy through bioassay

**Fig. 4:**
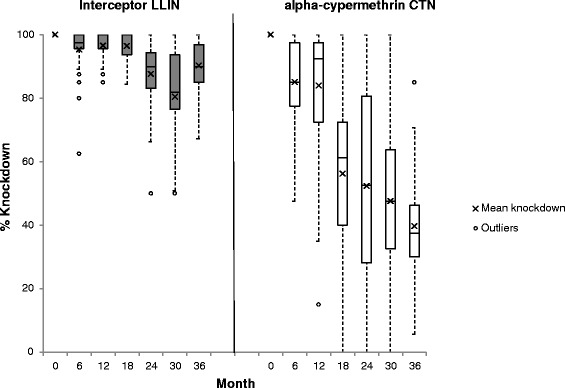
Median (IQR) and mean percentage *An. gambiae* (*s.s*.) (Kisumu) knockdown 1 h post-exposure to Interceptor LN and CTN pieces in cone bioassays

**Fig. 5:**
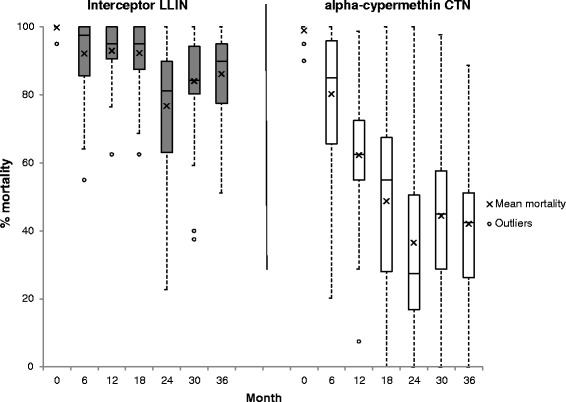
Median (IQR) and mean percentage *An. gambiae* (*s.s*.) (Kisumu) mortality 24 h post-exposure to Interceptor LN and CTN pieces in cone bioassays

**Fig. 6:**
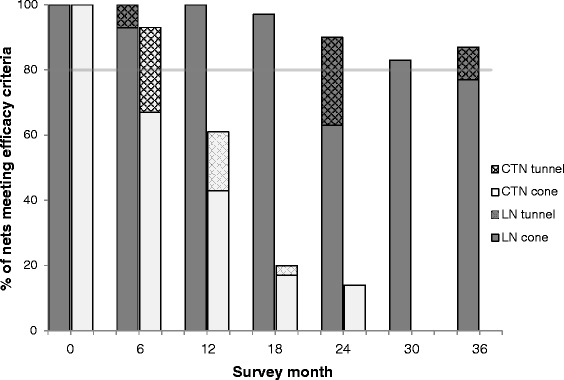
Percentage Interceptor LN & alpha-cypermethrin CTN meeting WHO efficacy criteria (solid bar = cone test, hatched bar = tunnel test^1^) by survey round. The horizontal line represents the acceptability cut-off for WHOPES full approval of the LN. No CTN passed at 30 or 36 months. ^1^WHO criteria: cone test: ≥ 80 % mortality and/or ≥ 95 % knockdown; tunnel test: ≥ 80 % mortality and/or ≥ 90 % blood feeding inhibition where control tunnel test > 35 % penetration into host chamber. Tunnel tests were carried out on nets that did not satisfy the cone test criteria

A total of 210 Interceptor LNs and 210 CTNs were sampled for bioassays and chemical analysis at 6 monthly intervals during the 3 years. Cone bioassay tests on Interceptor LN and CTN at baseline (before distribution) resulted in knock down of 100 % and mortality of > 99 % on all pieces tested (Figs. 4 and 5). After 6 months’ use the mean percentage mortality (± C.I.) was 92 % (88–96) on the Interceptor LNs and 80 % (74–87) on the CTNs (*t* = 5.25, *df* = 223, *P* = 0.0001; t-test) (Fig. 5). Similarly, knockdown was 95 % (92–98) on the Interceptor LNs compared to 85 % (80–90) on the CTNs (*t* = 6.03, *df* = 223, *P* = 0.0001; t-test) (Fig. 4). Two of the Interceptor LNs and 10 of the CTNs failed to meet the WHOPES criteria for the cone test. When the tunnel test was applied, all Interceptor LNs (100 %) and all but two of the CTNs (93 %, 28/30) met the WHOPES criteria (Fig. 6).

At 12 months, 97 % (29/30) of the Interceptor LNs but only 63 % (16/27) of the CTNs met the WHOPES criteria for cone and tunnel tests (Fisher’s exact X^2^ = 12.0, *df* = 1, *P* = 0.001). Only 3 % of Interceptor LNs failed the cone test but 56 % of CTNs failed the cone test at 12 months (Fig. 6). This difference between Interceptor LN and CTNs is also reflected in the mean percentage mortality of 93 % (90–96) for Interceptor LNs and 62 % (56–69) for the CTNs (*t* = 12.93, *df* = 223, *P* = 0.0001; *t*-test) in the cone bioassay tests (Fig. 5), and similarly in the percentage knockdown of 97 % (95–98) for the Interceptor LNs and 84 % (77–91) for the CTNs (*t* = 5.51, *df* = 223, *P* = 0.0001; t-test) (Fig. 4).

At 18 months, 97 % (29/30) of Interceptor LNs met the WHOPES criteria by either the cone or the tunnel test (Fig. 6). This figure declined to 90 % (27/30) at 24 months; at this sampling point fewer nets passed the cone bioassay criteria (63 %, 19/30) compared to before, but the majority (8/11) of nets that failed met subsequently the tunnel test criteria. At 30 and, crucially, 36 months Interceptor LN met the cone and tunnel test criteria with combined pass rates of 83 % (25/30) and 87 % (26/30) respectively; overall the incremental decrease in pass rate over the 36 months was small but significant (X^2^ for trend = 11, *df* = 1, *P* = 0.001). By contrast the efficacy of the CTN decreased considerably after 12 months, with only 20 % (6/30) meeting the criteria at 18 months, 14 % (4/30) at 24 months, and none at 30 or 36 months (X^2^ for trend = 125, *df* = 1, *P* = 0.0001). The major differences in the pass rates of Interceptor LN and CTNs after 1 year is also reflected in their percentage mortality and knockdown scores in cone bioassay tests between 12 months and 36 months (Figs. 4 and 5).

### Calibration of net efficacy and insecticide content in Phase III household trials and Phase II experimental huts trials

The Phase II experimental hut trial results of Interceptor LN and alpha-cypermethrin CTNs evaluated at the NIMR Amani Centre and presented in Table 5 were taken from Malima et al. [10]. The average alpha-cypermethrin content of Interceptor LNs and CTNs collected after 36 months in the present Phase III household trial was similar to the average alpha-cypermethrin contents of 20 times washed LNs and CTNs used in the Phase II experimental huts that led to the initial WHO interim recommendation for Interceptor LN (Table 5). Twenty times washed Interceptor LN (consistent with Interceptor LNs after 36 months field use) continued to demonstrate satisfactorily high levels of personal protection and mosquito mortality, both of which were significantly greater than the inadequate levels of protection and mortality recorded for 20 times washed CTNs. It follows that CTNs after 36 months field use would not provide adequate protection to users of such nets.

**Table 5:** Calibration of alpha-cypermethrin content and entomological outcomes in Phase II experimental hut trial (Malima et al. [10]) with alpha-cypermethrin content of nets in Phase III household randomised trial

	Interceptor LN	Interceptor LN	CTN
Number of washes in Phase II trial	0	20	20
% Mortality corrected for control^*^	92^a^	76^b^	44^c^
% Personal Protection^*^	79^a^	76^a^	6.4^b^
Mean concentration of alpha-cypermethrin (mg/m^2^) in Phase II trial	147	41	1.2
Mean concentration of alpha-cypermethrin (mg/m^2^) in Phase III trial	204^1^	42^2^	1.3^2^

^*^Percentages followed by the same letter superscript do not differ at 0.05 level

^1^at baseline before distribution

^2^after 36 months

### Adverse effects

Few adverse effects were reported by net users. At 12 months post-distribution, 8.4 % (21/249) of respondents recalled experiencing adverse effects during the first few days of use. The most common events were facial tingling (2 %), headache (1.6 %) and irritation (1.2 %). Adverse effects were slightly higher among users of the Interceptor LN compared to CTNs (11.5 % versus 5 %). Respondents reported that symptoms stopped once the net had been washed and nobody was deterred from using their nets. No adverse effects were reported in any of the subsequent surveys.

## Discussion

This WHOPES sponsored Phase III trial evaluated the efficacy of Interceptor LN over 36 months of household use using the standard WHO cone bioassay criteria of knockdown and mortality and the tunnel test criteria of mortality and blood feeding inhibition [5, 15]. At the conclusion of the trial 87 % of LNs sampled at 36 months met one or more of these efficacy criteria, and thus the LN product exceeded the 80 % threshold required to attain WHO full recommendation [20]. Each criterion contributed to determining whether a sampled batch of nets achieved the WHOPES threshold or not. For example at 36 months 30 % (9/30) of nets reached the threshold on the basis of both cone mortality and knockdown criteria, a further 40 % (12/30) passed on mortality criteria only (having failed on knockdown criteria), and 2/30 (7 %) passed on knockdown criteria only (having failed on mortality criteria). Thus it can be seen that cone mortality made by far the larger contribution to the overall pass rate. At 24 months the contribution of cone mortality was greater still: 63 % passed on the basis of cone mortality, 27 % passed on the basis of knockdown, but no nets passed on knockdown alone which means that knockdown rates made no contribution to the overall pass rate. A similar story emerged at 12 months with 93 % passing on the mortality criterion and only 3 % passing on the basis of knockdown alone. This indicates the major contribution of mortality over knockdown to the evaluation of pyrethroid LN efficacy.

Tunnel tests also made an important contribution. At 36 months 77 % of samples passed on the basis of cone criteria but critically an additional 10 % (3/30) passed on the basis of tunnel test criteria, lifting the overall pass rate to above the WHO threshold of 80 %. Tunnel tests can also be an important validation check on the veracity of the interpretation in the rare circumstances where anomalous results are recorded in the cone bioassays. For example, at 24 months an unexpectedly low 63 % of nets passed on the basis of cone criteria, but a further 27 % subsequently passed on the basis of tunnel test criteria.

At 36 months 26/30 (87 %) of LN had reached the required standard. It is sobering to reflect that had 3 LNs of this batch by chance not met the required standard Interceptor LN would have failed to reach the pass rate of 80 %. Just a few nets can exert great leverage around the 80 % threshold when only 30 nets form the basis of the decision. In response to this, WHOPES has decided to increase the sample size at the all important 36 month time point from 30 to 50 nets to improve statistical power and precision [5].

The CTNs were monitored beyond the anticipated 12 months’ end point because of theft of Interceptor LNs from the store. In-use CTN continued to be followed up for efficacy and chemical content. After 12 months’ use the insecticide content of the CTNs had decreased by 66 % relative to baseline; however, the majority of nets still met the efficacy criteria. From that point on the situation changed profoundly: after 24 months the insecticide content of the CTNs decreased by 94 % and few CTNs met the WHO efficacy criteria. Despite this, it is notable that while only a milligram per m^2^ alpha-cypermethrin residue remained on the average CTN, the nets still killed about 40 % of mosquitoes in cone bioassay. A similar observation was made during the Phase II experimental hut trial conducted in the same locality 3 years earlier; while after 20 standardised washes only a milligram of alpha-cypermethrin per m^2^ remained on the CTNs, mortality of 68 % was being recorded in cone bioassays and 44 % of free flying *An. gambiae* were still being killed by these nets in experimental huts [10]. However, the level of personal protection from mosquito biting from these nets was, at 6 %, insignificant both statistically and in terms of protection [10] and this provides a strong argument for always deploying LLIN over CTN.

The rate of loss of insecticide over time was more gradual in the Interceptor LNs, and was remarkably constant year by year. After 12 months of use the insecticide content of the LNs had decreased by 43 % of the initial content of 204 mg/m^2^, after 24 months it had decreased by a further 42 %, and after 36 months it had decreased by a further 38 %. At 36 months the average insecticide content was 42 mg alpha-cypermethrin per m^2^; this was remarkably similar to the 41 mg/m^2^ alpha-cypermethrin content observed in Interceptor LNs after 20 standardized washes in the Phase II experimental hut trials done in the same locality 3 years previously [10]. This similarity in chemical content between a Phase III household randomized trial and Phase II experimental hut trial indicates that the 20 standardized washes which LLINs undergo before testing in experimental huts is a fair approximation to the average loss of insecticide due to wear and tear, abrasion and washing that LLINs undergo during 3 years of household use. The outcomes of Phase II experimental hut trials would appear to be a reasonable prediction of the outcome of Phase III trials conducted in the community. While this correlation is encouraging, more LN products need to be evaluated and compared in Phase II experimental hut and Phase III household trials before this conclusion can be fully verified or justified. It is nevertheless encouraging – even fortuitous - that the arbitrary 20 washes that LLIN are purposefully subjected to in WHOPES Phase II seem a good approximation to Phase III after 3 years. In practice the number of washes that a net is subjected to during 3 years of household use may fall short of the 20 washes of Phase II; in the present Interceptor LN trial the average net was estimated to be washed 4.3 times a year or only 13 times over the 36 months. Under household use the average net would be subjected to more vigorous challenges than washing – the removal of surface insecticide through friction and abrasion in everyday use, for example - but over the 36 months this removal would seem to add up, or be equivalent to, the 20 washes of Phase II.

Taking the logic of the Phase II and Phase III calibration one step further, a typical Interceptor LN after 3 years of household use and alpha-cypermethrin content of 42 mg/m^2^ should, as predicted by experimental hut trials, continue to kill up to 78 % of hosting seeking *An gambiae* that contact the net and would still provide 76 % protection to the occupants [10]. Given the major loss of efficacy and protection observed with the average CTN after 3 years, discussed above, this concludes the argument for always deploying LLIN over CTN.

At all time points, trial participants reported a high frequency of net use all year round; this assertion was corroborated by the high proportion of nets observed hanging above the beds. After 36 months in the field most nets had incurred damage: few were without holes (only 17 %) and most were dirty or very dirty (70 %). The Tanzanian nets were in worse condition than the Interceptor LNs studied in Uganda where after 36 months, 27 % were without holes and 29 % were scored as dirty or very dirty [13]. The bioefficacies were very similar between the Tanzanian and Ugandan studies, and therefore the accumulation of dirt or soot on the nets may not affect the toxicity of the pyrethroid, as noted by Kayedi et al. [21]. Nevertheless the trials do highlight the issue of durability and the importance of high denier netting to achieve that durability (the Interceptor LN issued were only 75 denier). The majority of holes were size 1 and most were found on the lower half of the nets where abrasion caused by tucking under the mattress was more likely to occur. While the attrition of nets (loss of nets from households) was not monitored, the trend in hole index over time indicates that it stabilized after 24 months. Nets were probably being discarded once they had become highly holed so the residual population of nets maintained a more regular pHI after 24 months. A ‘steady state’ hole index after 24 months’ use has been observed during sequential household surveys in the Phase III evaluations of PermaNet 2.0 and Olyset LN [22–24]. A threshold hole index or hole area which a given proportion of LN are expected to reach after 3 years are important criteria for WHOPES to establish and encourage manufacturers to improve the durability and longevity of their products.

Although some adverse effects were reported during the first weeks of net usage, these were rare and short lived and did not deter Interceptor LN use.

The Tanzanian trial was one of three trials commissioned by the WHO Pesticide Evaluation Scheme [20], while a fourth non-WHOPES trial was conducted independently in Uganda [13]. After 36 months, the percentage of nets that met the WHO efficacy criteria was 98 % at one site in India (Gujarat), 73 % at a second site in India (Chhattisgarh) and 83 % in Uganda. The data from Gujarat had to be discounted as the majority of Interceptor LN exceeded the tolerance limit of alpha-cypermethrin content at baseline and the study in Chhattisgarh failed after 36 months as only 73 % of the nets passed the threshold bioassay criteria [25]. In the two trial locations where the Interceptor LNs were within the acceptable range for alpha-cypermethrin content – Tanzania and Uganda – the LNs did meet the WHOPES efficacy criteria after 3 years of use [20].

## Conclusions

In this WHOPES Phase III household randomized trial conducted in Tanzania, Interceptor LN succeeded in meeting the WHOPES efficacy criteria for long-lasting insecticidal nets after 36 months of use. On the basis of this trial and one other non WHOPES trial where the LNs were within the acceptable range of alpha-cypermethrin content at baseline, Interceptor LN obtained WHO full recommendation. The calibration of Interceptor LNs at 36 months and Interceptor LNs with similar levels of alpha-cypermethrin content tested in Phase II experimental hut studies predicts that such nets would continue to give high levels of personal protection and mosquito control after 3 years of household use provided net integrity is maintained. Threshold net integrity criteria that a LN should reach in order to obtain recommendation should be established by WHOPES to improve LN durability.
